# Rapid detection of functional gene polymorphisms of TLRs and IL-17 using high resolution melting analysis

**DOI:** 10.1038/srep41522

**Published:** 2017-02-02

**Authors:** Johanna Teräsjärvi, Antti Hakanen, Matti Korppi, Kirsi Nuolivirta, Kirsi Gröndahl-Yli-Hannuksela, Jussi Mertsola, Ville Peltola, Qiushui He

**Affiliations:** 1 Department of Medical Microbiology and Immunology, University of Turku, Turku, Finland; 2 TYKS-Sapa, Microbiology and Genetics Service Area, Microbiology Branch, Turku University Hospital, Turku, Finland; 3 Center for Child Health Research, Tampere University and University Hospital, Tampere, Finland; 4 Department of Pediatrics, Seinäjoki Central Hospital, Seinäjoki, Finland; 5 Department of Pediatrics and Adolescent Medicine, Turku University Hospital, Turku, Finland; 6 Department of Medical Microbiology, Capital Medical University, Beijing, China

## Abstract

Genetic variations in toll-like receptors (TLRs) and IL-17A have been widely connected to different diseases. Associations between susceptibility and resistance to different infections and single nucleotide polymorphisms (SNPs) in TLR1 to TLR4 and IL17A have been found. In this study, we aimed to develop a rapid and high throughput method to detect functional SNPs of above mentioned proteins. The following most studied and clinically important SNPs: TLR1 (rs5743618), TLR2 (rs5743708), TLR3 (rs3775291), TLR4 (rs4986790) and IL17 (rs2275913) were tested. High resolution melting analysis (HRMA) based on real-time PCR combined with melting analysis of a saturating double stranded-DNA binding dye was developed and used. The obtained results were compared to the “standard” sequencing method. A total of 113 DNA samples with known genotypes were included. The HRMA method correctly identified all genotypes of these five SNPs. Co-efficient values of variation of intra- and inter-run precision repeatability ranged from 0.04 to 0.23%. The determined limit of qualification for testing samples was from 0.5 to 8.0 ng/μl. The identical genotyping result was obtained from the same sample with these concentrations. Compared to “standard” sequencing methods HRMA is cost-effective, rapid and simple. All the five SNPs can be analyzed separately or in combination.

Toll-like receptors (TLR) are a critical part of innate immunity. So far, ten functional TLRs have been identified in humans. TLRs recognize a wide range of pathogen-associated molecular patterns (PAMPs) from microorganisms and danger-associated molecular patterns (DAMPs) released from damaged tissue^1^,^2^. In turn, interleukins (ILs) are a large group of immune modulating proteins that bind to their specific receptors and play an important role in the communication in the immune system^3^.

TLR2 and TLR1 are members of the TLR2 subfamily which comprises TLR1, TLR2, TLR6 and TLR10. TLR1 is co-receptor of TLR2 and they occur as heterodimers on a cell surface. TLR1 and TLR2 are capable of detecting a wide range of ligands, especially from Gram-positive bacteria and mycobacteria. The main ligand of TLR2 is peptidoglycan and that of TLR1 is triacylated lipopeptide. TLR3 is located on the surface of endosome and it recognizes viral dsRNA, synthetic oligonucleotides and DAMPs. TLR4 is located on the cell surface or on the surface of endosome. It recognizes lipopolysaccharides (LPS) and PAMPs of Gram-negative bacteria^4^. Both TLR3 and TLR4 are also innate antiviral immune response receptors. IL17 (IL17A) is a pro-inflammatory cytokine. It is produced by different types of cells, including CD4+ T cells and neutrophils. IL17 orchestrates for recruitment of neutrophils and stimulates production of the antimicrobial peptides^3^.

Genetic variations, especially single nucleotide point mutations (SNPs) can affect development of disease in individuals and responses to pathogens, drugs, vaccines and other agents. Recent studies have shown a number of SNPs in genes coding the TLR1, TLR2, TLR3, TLR 4 and IL17A. These SNPs have been associated with susceptibility or resistance to different infections^4^,^5^. In this study, we took into account the most studied and clinically important SNPs of the TLRs and IL17, which are listed in Table 1.

One of the most studied SNPs in *TLR2* gene is 2258G > A (rs5743708). This SNP causes amino acid substitution from arginine (Arg) to glutamine (Gln) in the C-terminal end of the TLR2 at position of 753. Individuals who carry the minor allele A, may have increased risk for atopy^6^, sepsis^7^,^8^ and susceptibility to tuberculosis^9^,^10^. Haerynck and his colleagues have found a correlation between allele A and faster decline of forced expiratory volume 1 (FEV1) result of patients with cystic fibrosis^11^. Schröder *et al*. have shown that heterozygous polymorphism (Arg753Gln) can impair immune activation by *Borrelia burgdorferi* and may protect from late stage Lyme disease^12^. The SNP 1805G > T (rs5743618) in *TLR1* gene leads to substitution of amino acid at the position 602 from serine (Ser) to isoleucine (Ile) on the cytoplasmic side of the transmembrane domain of the receptor. It affects the cell surface trafficking in several cell types^4^. Allele G, which is a minor allele in all other populations, except Europeans, is associated with increased susceptibility to asthma and allergic rhinitis after hospitalization due to bronchiolitis in infancy^13^ and is also a risk factor of tuberculosis^14^,^15^,^16^. The SNP 1234C > T (rs3775291) in *TLR3* gene causes an amino acid change from leucine (Leu) to phenylalanine (Phe) at the position 412 in the extracellular domain of this protein. The dominant allele C was found to be a risk factor for tick-borne encephalitis virus (TBEV)^17^,^18^,^19^ and bronchiolitis in early infancy^20^. TLR4 SNP 896A > G (rs4986790) causes an amino acid change from aspartic acid (Asp) to glycine (Gly) at the position of 299. It is located in the extracellular domain of this protein. In the previous studies we have shown an association between the minor allele G and increased risk of repeated colonization of *Moraxella catarrhalis*^21^. The allele G has also been connected to an increased risk of different disorders caused by bacteria and viruses, such as septic shock due to infection by Gram-negative bacteria^22^,^23^, increased mortality of children with invasive meningococcal disease^24^, chronic cavitary pulmonary aspergillosis^25^ and susceptibility to respiratory syncytial virus (RSV) infection^26^. The IL17A SNP −197G > A (rs2275913) is located in the promoter of *IL17A* gene and has an effect on its transcriptional regulation. Children who carry genotype AA of IL17A have usually low serum level of IL17A. We have previously reported an association between the minor allele A and increased colonization of *Streptococcus pneumoniae* in young Finnish children^27^. Other studies have shown associations of the allele A with gastrointestinal diseases^28^ and childhood asthma^29^. The SNPs studied in TLRs and IL17A and potential effects on susceptibility and resistance to different diseases are summarized in Table 1.

Direct DNA sequencing is considered a “golden standard” for identification of the SNPs. However, the melting properties of DNA have enabled the development of many rapid and high throughput methods^30^, high resolution melting analysis (HRMA) being one of them. The method is based on analysis of released fluorescence from binding dye of the double stranded DNA (dsDNA) and is proven to be a simple, relatively rapid and low-cost tool for SNP scanning. PCR is the first step of HRMA followed by binding of a dye. The dye binds to dsDNA, when amplicons are denatured and then rapidly re-annealed. Melting analysis starts when dsDNA amplicons with saturated dye are gradually heated and release the fluorescence dye. The data can then be analyzed based on the melting temperature (Tm) and on the shape of the normalized melting curves. HRMA is ideal for clinical use because PCR amplification and melting curve analysis can be performed on the same plate and without any post-PCR processing. This saves time and decreases the risk of contamination.

HRMA method has been used for genotyping of *Yersinia enterocolitica*^31^, for screening of oncogene mutations^32^ and for identifying of common variants in methylenetetrahydrofolate reductase (*MTHFR*) gene^33^. A meta analysis has shown that HRMA method is a highly sensitive, simple and low-cost test to detect human disease-associated mutations, especially for samples with mutations of low incidence^34^. Recently HRMA method has been applied for detection of TLR1 (rs5743618), TLR2 (rs5743708) and TLR4 (rs4986790) SNPs^35^,^36^,^37^.

In our previous studies, we have used PCR-based pyrosequencing, Sanger sequencing and Sequenom massARRAY iPlex Gold method for detecting the functional SNPs of TLRs^21^ and IL17^27^. These methods are generally time consuming and expensive, especially for a large number of samples. Increased knowledge of function and importance of TLRs and ILs have aroused the interest in clinicians to variations of functional SNPs. In this present study we aimed to develop a high throughput method based on HRMA for rapid detection of TLR1 (rs5743618), TLR2 (rs5743708), TLR3 (rs3775291), TLR4 (rs4986790) and IL17A (rs2275913). The knowledge of the genetic variations in these important molecules will increase our understanding of why some individuals have higher likelihood of infections and different outcomes after the diseases. In the future this knowledge may help plan a more personalized care chain.

Normally, the validation and verification of a method follow ISO standards or Organization for Economic Cooperation and Development guidelines. For HRMA validation there are no such standards available. We used selected validation parameters, which are described in previous literature^30^,^33^.

## Results

### PCR and HRM parameters optimization

Three samples with known genotypes were used to determine the proper concentration of MgCl_2_ and annealing temperature (T_a_ °C) for each individual assay, shown in Table 2. In order for the method to be suitable for diagnostic use it would be necessary to analyze all the SNPs on one plate during the same run. Therefore, the second step on the optimization process was to find out the optimal annealing temperature (T_a_) which works for all used primer sets. The optimal T_a_ varied between 60.2 °C and 57 °C in individual assays. Two test runs were made and the annealing temperature was set at 58 °C on the first run and at 59 °C on the second run. When comparing the annealing curves and melting profiles, the higher annealing temperature gives more representative results, thus 59 °C is used as the annealing temperature in the assay.

### Validation

**Figure 1:**
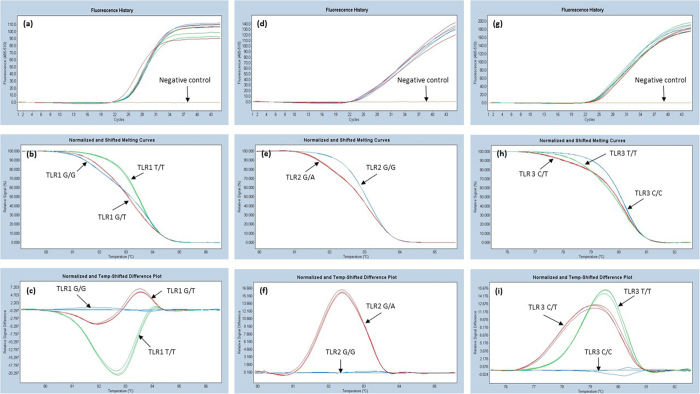
Analysis of TLR1 SNP 1805G > T (rs5743618), TLR2 SNP 2258 G > A (rs5743708) and TLR3 SNP 1234C > T (rs3775291). The upper three pictures represent PCR amplification of TLR1 (**a**), TLR2 (**d**) and TLR3 (**g**). Normalized melting profiles of the SNPs are shown in middle three pictures (**b,e** and **h**). In the picture h can perceive how both homozygote genotypes (TLR3 C/C and T/T) forms similar normalized and shifted melting curve and heterozygous melting curve (TLR3 C/T) settles between the homozygote curves. The last pictures (**c,f** and **i**) represent normalized and temp-shifted difference plot of the TLR1, TLR2 and TLR3 SNPs. In figure c TLR1 SNP (rs5743618) major genotype in Finnish population, was chosen to be as a baseline and in figures f and i wild type genotype was chosen to be as a baseline.

**Figure 2:**
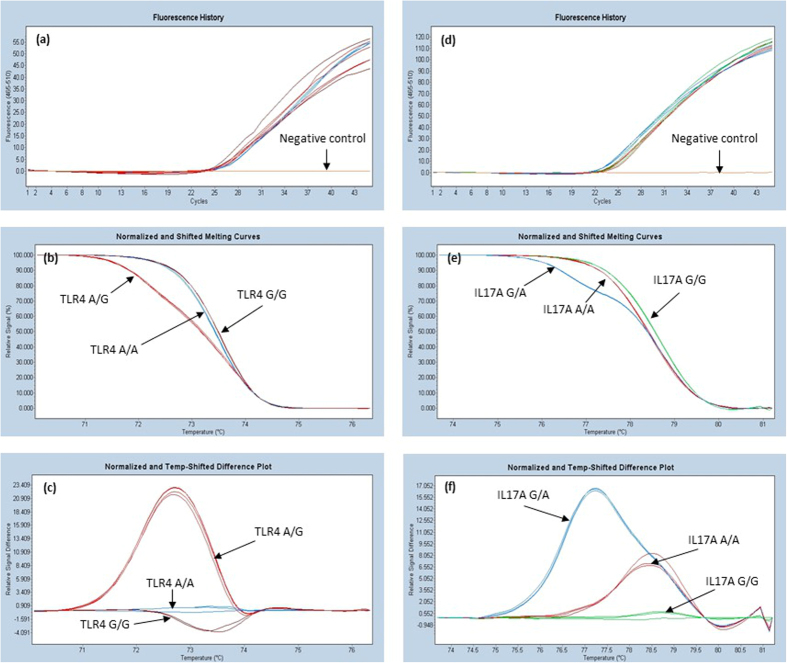
Analysis of TLR4 SNP 896A > G (rs4986790) and IL17 SNP − 197G > A (rs2275913). The first pictures represent annealing step of TLR4 (**a**), and IL17 (**d**) PCR. The normalized melting profiles of the SNPs are shown in the two latter pictures (**b** and **e**). The last pictures (**c** and **f**) represent normalized and temp-shifted difference plot of the TLR4 and IL17 SNPs. Wild type genotype was chosen to be as a baseline.

Based on the optimization runs, two samples with the best melting curve, were chosen to represent each variant and later these samples were used as the controls. Melting curves of different genotypes of TLR1, TLR2 and TLR3 used as controls are shown in Fig. 1 and those of TLR4 and IL17 are shown in Fig. 2. Sequences of the controls were confirmed by Sanger sequencing (data not shown).

Total of 77 samples were used to test the method accuracy of TLR1 (rs5743618), TLR2 (rs5743708) and TLR3 (rs3775291). The results obtained by HRMA were compared to pyrosequencing results. Resemblance between HRMA and sequencing genotyping results was 100%. Total of 60 samples were used to test the method accuracy of TLR4 (rs4986790) and IL17 (rs2275913). The results obtained by HRMA were compared to those by pyrosequencing (TLR4) and Sequenom massARRAY iPlex Gold method (IL17). Identical genotyping results were obtained.

For analysis of melting curves, all tested samples were grouped together with regard to the controls, and melting profiles were identical for DNA samples with the same genotype. The coefficient of variation (CV) of intra- and inter-run precision-repeatability varied between 0.04 and 0.23%. In TLRs and IL17A genotypes there were no considerable differences in CV% of melting profiles.

**Figure 3:**
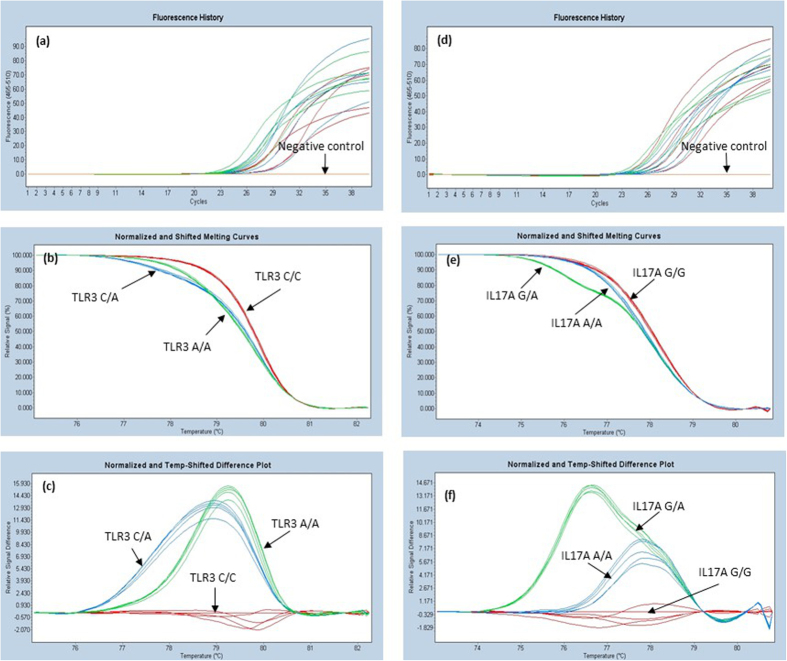
Effect of the template DNA concentration on the annealing curves and melting profiles of the amplicons.

The limit of quantification was tested for the five studied SNPs using a DNA template. Two fold serial dilutions of the sample were used, ranging from 0.5 to 8.0 ng/μl. The identical genotyping result was obtained from the same sample with different concentrations for all five studied SNPs. Only a marginal change in melting curve profiles and melting temperatures was observed between different DNA concentrations of the same sample (Fig. 3).

### Confirmation of TLR1 SNP

**Figure 4:**
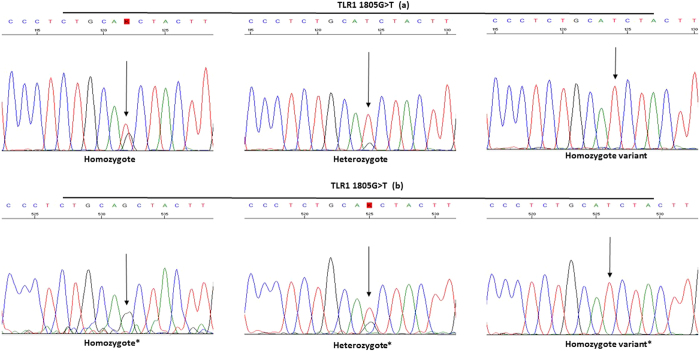
TLR1 SNP 1805 G > T (rs574318) sequence before and after the pre-PCR. In the upper-part of the picture (**a**) is clearly to seen how the nucleotide T from TLR6 overlaps with TLR1 1805G > T (arrow). In the lower part of the figure (**b**) is shown the TLR1 SNP sequence after the pre-PCR step. After the pre-PCR step the HRMA primer set amplified only to TLR1 and the T-peak from the TLR6 SNP is not perceived in the sequence (arrows in lower part of figure).

**Figure 5:**
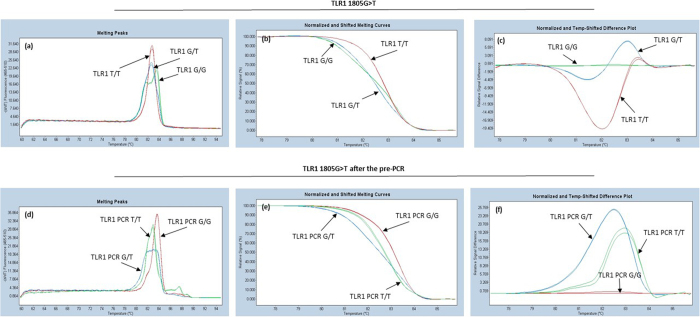
Melting Peaks (**a** and **d**), Normalized and Shifted melting Curves (**b** and **e**) and Normalized and Temp-Shifted Difference Plots (c and f) before and after the pre-PCR step. In both cases, before and after the pre-PCR step HRMA is capable to recognize all three genotypes. The curves before pre-PCR step are atypical (**a**,**b** and **c**), homozygote genotype GG exist like heterozygote variant. In lower part of the figure (**d**,**e** and **f**) curves are typical for each genotype.

At the beginning of this study we noticed that HRMA melting curves and sequencing diagrams were atypical for the TLR1 (rs5743618). HRMA clearly divided the six testing samples with known three genotypes (two for each wild type, heterozygote variant and homozygote variant) in three different groups (Fig. 5a–c). Although the sequencing diagram (Fig. 4a) showed that there was a nucleotide T-peak in each sample, even in those which were supposed to be the homozygous wild type G genotype. We thus performed a Genbank searching and found that TLR1 primer set used for HRMA was not specific only for TLR1 rs5743618 (1805G < T), but the primers were also capable of binding to *TLR6* gene. The closer examination uncovered a ~300 bp area in TLR1, with 97% similarity to TLR6. The background peak T was actually derived from co-amplification of the *TLR6* and *TLR1* genes. In order to differentiate the three genotypes of TLR1 rs5743618 we redesigned a set of primers only specific to TLR1. The background peak T in the TLR1 sequence disappeared after the pre-PCR step (Fig. 4b) and both normalized HRMA curves became typical (Fig. 5e,f). The three genotypes of TLR1 rs5743618 (1805G < T) were clearly distinguished by HRMA no matter whether the pre-PCR step was performed or not (Fig. 5).

## Discussion

The purpose of the study was to develop a rapid, less expensive and high throughput genotyping method for functional polymorphism of important innate immune genes such as TLRs and IL-17. A set of samples were selected from two of our earlier studies: bronchiolitis and STEPS studies conducted in Finland in which functional SNPs of TLRs and IL-17 were analyzed to find out their associations with development of subsequent infections and post-bronchiolitis outcomes^13^,^20^,^21^,^27^,^38^. The genotyping method was based on real-time PCR combined with HRMA. The results were compared to the PCR-based sequencing methods such as pyrosequencing and Sanger sequencing. All studied genotypes of these four TLRs and IL-17A were correctly identified with HRMA. Compared to the “traditional” sequencing methods HRMA is cost-effective, rapid and simple. All the five SNPs can be analyzed separately or together which make it well-adapted analysis tool for diagnostic use.

In general, post-PCR melting analyses are used to separate homozygote and heterozygote variants. Separation between homozygote wild type and homozygote variants is more difficult, because they differ only in between melting temperatures (T_m_) but the shapes of normalized and shifted melting curves are generally similar (Fig. 1h). In our case, the difference between a homozygote wild type and a homozygote variant is >0,5 °C. These types of SNPs are relatively easy to be differentiated, but when analyzing all the SNPs on the same plate at the same time, PCR and melting conditions require certain compromises. In this case, the annealing conditions of PCR are not fully optimized for all the SNPs. In our study, annealing temperature was optimal for IL-17 and TLR4 (Table 2). Because of this, the shapes of the melting curves for the TLR1–3 were not as sharp and representative as they were when tested separately. However, simultaneous amplifications of the five PCRs were proven to be specific and we were able to distinguish genotypes of all these five SNPs. Moreover, both intra- and inter-run precision-repeatability CV were very low (<0,2%), showing that HRMA is a reliable method for the SNP analysis of TLR1-4 and IL17A.

TLR1, TLR2, TLR6 and TLR10 comprise the TLR2 subfamily. These TLRs have similarities in their structure and sequence. The sequence similarities may become a problem when using HRMA to determine the TLR SNPs of this subfamily. We met this kind of problem with TLR1 SNP rs5743618. The SNP is located in the middle of ~300 bp area with 97% similarity to *TLR6* gene. We used the pre-PCR step to confirm the identity of each TLR1 variant. In the *TLR6* gene there is also a SNP (rs62622399) in the same location as *TLR1* SNP rs5743618. The SNP in *TLR6* gene has been found only in one small Banthu population in Africa. Since TLR6 SNP rs62622399 is extremely rare (only reported in two cases) and the pre-PCR step is quite laborious for the routine diagnostic test, TLR1 SNP rs5743618 can be analyzed without the pre-PCR step, as clearly shown in Fig. 5.

Based on the previous studies^39^,^40^,^41^ it is known that high-resolution DNA melting analysis has some limitations but in this study we have taken account these limitations and shown that HRMA is a reliable and accurate method for the SNP analysis of TLR 1-4 and IL17A. The best results are obtained when the control DNAs are of high-quality and the concentrations of these control DNAs and studied samples are closed to each other.

It is known that quantitative PCR is prone to have variation in the yield of amplified products, when different concentrations of target DNAs are used. In this present study, two-fold serial dilutions of a DNA sample ranging from 0.5 to 8.0 ng/μl were used to test the effect. For all five studied SNPs, the identical genotyping result was obtained from the same DNA sample with different concentrations. Only a marginal change in melting curve profiles and melting temperatures was observed between different DNA concentrations of the same sample (Fig. 3). However, it should be kept in mind that the difference in DNA concentrations tested was only less than 16 folds.

TLRs and IL-17 are key molecules in innate and adaptive immunity. Genetic variations in TLRs and ILs have been widely connected to different diseases like cancer, autoimmunity disorders and infections. Furthermore, many studies have shown the associations between infections and SNPs in TLR2, TLR3, TLR4 and IL17A. These SNPs have been associated with susceptibility or resistance to different infections^3^,^4^,^5^. The methods developed in this study could be used for screening of patients with different disease conditions and the information will increase our understanding about the connections between different diseases and the SNPs and could provide a tool for clinicians to design more effective precision medicine.

## Materials and Methods

### Samples and DNA extraction

Total of 113 anonymously selected samples from two of our earlier studies^13^,^20^,^21^,^27^ were used in this study. The first 53 DNA samples were isolated from the whole blood samples which were collected from full-term infants hospitalized for bronchiolitis at <6 months of age during the period December 1, 2001 through May 31, 2002 and during the period from October 28, 2002 through May 31, 2004^13^. These samples were stored at −70 °C until DNA extraction. Rest of 113 samples were randomly selected DNA samples from a Finnish birth cohort study, called “Steps to the healthy development and well-being of children” (the STEPS study)^38^. The blood samples of the STEPS Study were taken between August 2008 and June 2010, when healthy infants visited the study clinic at the age of 2.6 months. These samples were immediately transported to the laboratory, where the DNA extraction was performed. The DNA samples are stored at −20 °C. The protocols of the two studies were approved by the Ethics Committees of Tampere University Hospital District, Tampere and of the Hospital District of Southwest Finland, Turku, Finland. The parents of participating children provided their written, informed consent. The personal data of the study subjects were not given to the laboratory performing the genetic studies. The methods were carried out in accordance with the relevant guidelines, including any relevant details.

In both cases, 200 μl of the whole blood was used for DNA isolation. Extraction was done with QIAGEN QIAamp DNA Blood Mini Kit 250 (Qiagen, Hilden, Germany) according to the manufacturer’s protocol. DNA quality and concentration were measured by a spectrophotometer (NanoDrop ND-1000, Thermo Scientific, Waltham, Massachusetts, USA). The above mentioned five SNPs of TLR1, TLR2, TLR3 and IL17A were detected and the results have been previously published^13^,^20^,^21^,^27^,^38^.

### Primer design

To maximize the difference between melting peaks (T_m_) in variant genotypes, primers were designed to define a small fragment (<150 bp) surrounding the SNP in interest. The manufacturer’s recommendations for CG (%) content and primer melting temperatures were taken into account. Primers (Table 2) were designed with Primer-Blast design tool (http://www.ncbi.nlm.nih.gov/tools/primer-blast/).

TLR1 HRMA primers were set specific for SNP rs5743618 (1805G < T), which is located in the middle of ~300 bp area with 97% similarity to TLR6. In order to separate the two TLRs, a Pre-PCR was used to confirm specific annealing and melting in HRMA. The sequences of the two primers used in the TLR1 pre-PCR were forward: 5′-GAAAGGAGACTGTTCTTGG-3′ and reverse: 5′-CTTTTGAGCTTGTGATAACTGCTAG -3′.

All primers were ordered from Sigma-Aldrich Company (Saint Louis, Missouri, USA). The Primers used in HRMA were with HPCL quality.

### Optimization of PCR and HRM parameters

All HRM analyses were performed by LightCycler480 version 5.1 (Roche, Basal, Switzerland). Roche’s original melting master kit (LightCysler 480 High Resolution Melting Master) and 96-well plates (LightCycler 480 Multiwell 96, White) were used. Three samples with known genotypes were used to determine the proper concentration of MgCl_2_ and annealing temperature (T_a_°C) for each individual assay (Table 2). Serial dilutions from 1 to 3.5mM of MgCl_2_ were tested. Determining T_a_°C was carried out by a gradient PCR. In each run reaction volume was 20 μl consisting of 3 μl genomic DNA (8.0 ng/μl) and 17 μl of master mix including 10 μl melting master dye with the additional concentration of MgCl_2_ (serial dilution) and 0.2 μM of forward and reverse primers. The gradient PCR reaction program started with an initial denaturation of 10 min at 95 °C followed by 45 cycles amplification of 10s at 95 °C and annealing temperature gradient from 57 °C to 62 °C and 15 s at 72 °C. After the PCR step, HRM melting cycle conditions were as outlined by Roche: first heated to 95 °C and held for 1 min, cooled to pre-hold temperature (40 °C) followed by melting interval for collecting fluorescence from 60 °C to 95° at ramp rate of 0.2 °C per second.

For TLR1 optimization both pure DNA and the PCR product from pre-PCR were used. The pre-PCR step was performed with Applied Biosystems Thermal Cycler (Thermo Scientific, Waltham, Massachusetts, USA), using the following parameters; denaturation 10 min at 94 °C, 40 cycles at 94 °C, annealing at 55 °C for 40s and 72 °C for 60s, followed by one cycle at 72 °C for 5 min.

The second step in the optimization process was to find out the optimal conditions where all the SNPs can be run on the same plate at the same time. Optimal MgCl_2_ concentrations are shown in Table 2. Optimal T_a_ varied between 57 °C and 60.2 °C. Two test runs were made. PCR and HRM conditions were the same as described previously, except the annealing temperature, which was on the first run 58 °C and on the second run 59 °C.

Agarose gel (1.5%) electrophoresis was used to confirm specific PCR amplifications.

### Validation of HRM parameters

A total of 77 DNA samples were analyzed for TLR1–3 and 60 samples for TLR4 and IL17A SNPs (Table 2) by HRMA. Based on HRMA melting profiles of the SNPs two samples were selected to represent of each genotype. The samples were sent to the Finnish Institute for Molecular Medicine, Helsinki, Finland for sequencing to confirm the identity of the sequence. These samples were later used as the controls in each run.

Before the High Resolution Melting analysis, all samples were genotyped with an additional method, either pyrosequencing^21^, or Sequenom massARRAY iPlex Gold platform (IL17A)^27^. Resemblance between melting analysis and sequencing was determined and presented in % concordance.

Intra-assay precision reflects variability among replicate determinations within the same assay run. In turn, inter-run precision is the comparison of the results between the series run on different days. The same control samples were used in both assays. For intra-run assay the samples were prepared in five replicates at one concentration level in one trial run and for the inter-run assay the same DNA samples were analyzed in one trial performed on three different days using freshly prepared reagents. Intra- and inter-run precision-repeatability was presented as coefficient of variation (%).

Limit of Quantification was detected using a DNA template dilution ranging from 0.5 to 8.0 ng/μl.

## Additional Information

**How to cite this article**: Teräsjärvi, J. *et al*. Rapid detection of functional gene polymorphisms of TLRs and IL-17 using high resolution melting analysis. *Sci. Rep.* **7**, 41522; doi: 10.1038/srep41522 (2017).

**Publisher's note:** Springer Nature remains neutral with regard to jurisdictional claims in published maps and institutional affiliations.

## Figures and Tables

**Table 1:** Function of the studied SNPs and the allele frequencies reported in Finnish population (1000 Genome project) and in this study.

Target	SNP id	Nucleotide change	Amino acid change	Allele Frequency (1000 Genome project)*	Allele Frequency of this study	Potential effect and reported associations
TLR1	rs5743618	1805G > T	Ser > Ile	A: 0.172 C: 0.828	A: 0.208 C: 0.792	Compromised signaling; Asthma, Allergic rhinitis and TB
TLR2	rs5743708	2258G > A	Arg > Gln	G: 0.970 A: 0.030	G: 0.948 A: 0.052	Compromised signaling; Sepsis, TB, Atopy, Cystic Fibrosis decline
TLR3	rs3775291	1234C > T	Leu > Phe	C: 0.667 T: 0.333	C: 0.578 T: 0.422	Compromised signaling; Tick-borne encephalitis virus (TBE), Bronchiolitis
TLR4	rs4986790	896A > G	Asp > Gly	A: 0.884 G: 0.116	A: 0.848 G: 0.155	Hyporesponsiveness; *M.catarrhalis* and *H.influenzae* colonization, Septic shock, Respiratory syncytial virus (RSV)
IL17A	rs2275913	−197G > A	—	G: 0.571 A: 0.429	G: 0.530 A: 0.470	Reduced production; *S.pneumoniae* colonization, Childhood asthma, Gastrointestinal diseases

^*^Reference 42.

**Table 2:** Primers and parameters used in the High Resolution Melting Analyze in each individual assay*.

Target	SNP id	Primers	Product size (bp)	Annealing temperature (°C)	MgCl_2_ concentration (mM)
TLR1	rs5743618	F:5′-CTGGCACACCATCCTGAGAT-3′R:5′-GTTGGCTGTGACTGTGACCT-3′	70	57	2.5
TLR2	rs5743708	F:5′-TCTCAATTCTTCTGGAGCCCAT-3′R:5′-GGCCACTCCAGGTAGGTCT-3′	96	57	2.5
TLR3	rs3775291	F:5′-ACTTGCTCATTCTCCCTTACACAT-3′R:5′-GCCCAATTTCATTAAGGCCCA-3′	125	60	3
TLR4	rs4986790	F:5′-ACCATTGAAGAATTCCGATTAGCA-3′R:5′-CCAGGGAAAATGAAGAAACATTTG-3′	95	59	2.5
IL17A	rs2275913	F:5′-TCTGCCCTTCCCATTTTCCTTC-3′R:5′-GGTTAAAATTTCCGCCCCCAATT-3′	70	59	3

^*^Annealing temperature 59 °C was used when analyzing all the five SNPs in same run.
